# Diagnostic yield of rare skeletal dysplasia conditions in the radiogenomics era

**DOI:** 10.1186/s12920-021-00993-0

**Published:** 2021-06-06

**Authors:** Ataf H. Sabir, Elizabeth Morley, Jameela Sheikh, Alistair D. Calder, Ana Beleza-Meireles, Moira S. Cheung, Alessandra Cocca, Mattias Jansson, Suzanne Lillis, Yogen Patel, Shu Yau, Christine M. Hall, Amaka C. Offiah, Melita Irving

**Affiliations:** 1 Department of Clinical Genetics, Guy’s and St Thomas’ NHS Foundation Trust, London, UK; 2 College of Medical and Dental Sciences, University of Birmingham, Birmingham, UK; 3 The Royal Wolverhampton NHS Trust, Wolverhampton, UK; 4 Radiology Department, Great Ormond Street Hospital for Children, NHS Foundation Trust, London, UK; 5 Clinical Genetics Department, University Hospitals Bristol and Weston, Bristol, UK; 6 Department of Paediatric Endocrinology, Evelina London Children’s Hospital, London, UK; 7 Viapath LLP, Guy’s Hospital, 5th Floor Tower Wing, London, SE1 9RT UK; 8 Neurogenetics, Rare and Inherited Disease Laboratory, North Thames GLH, Barclay House, 37 Queen Square, London, WC1N 3BH UK; 9 Great Ormond Street Hospital for Children, London, UK; 10 Emeritus Professor of Paediatric Radiology, Institute of Child Health, University of London, London, UK; 11 Academic Unit of Child Health, University of Sheffield, Sheffield, UK; 12 Division of Genetics and Molecular Medicine, King’s College London School of Medicine, London, UK

**Keywords:** Mendelian, Molecular genetic test, Monogenic, Next-generation sequencing, Skeletal dysplasia, Exome sequencing, Genome sequencing, Yield

## Abstract

**Background:**

Skeletal dysplasia (SD) conditions are rare genetic diseases of the skeleton, encompassing a heterogeneous group of over 400 disorders, and represent approximately 5% of all congenital anomalies. Developments in genetic and treatment technologies are leading to unparalleled therapeutic advances; thus, it is more important than ever to molecularly confirm SD conditions. Data on ‘rates-of-molecular yields’ in SD conditions, through exome sequencing approaches, is limited. Figures of 39% and 52.5% have been reported in the USA (n = 54) and South Korea (n = 185) respectively.

**Methods:**

We discuss a single-centre (in the UK) experience of whole-exome sequencing (WES) in a cohort of 15 paediatric patients (aged 5 months to 12 years) with SD disorders previously molecularly unconfirmed. Our cohort included patients with known clinical diagnoses and undiagnosed skeletal syndromes. Extensive phenotyping and expert radiological review by a panel of international SD radiology experts, coupled with a complex bioinformatics pipeline, allowed for both gene-targeted and gene-agnostic approaches.

**Results:**

Significant variants leading to a likely or confirmed diagnosis were identified in 53.3% (n = 8/15) of patients; 46.7% (n = 7/15) having a definite molecular diagnosis and 6.7% (n = 1/15) having a likely molecular diagnosis. We discuss this in the context of a rare disease in general and specifically SD presentations. Of patients with known diagnoses pre-WES (n = 10), molecular confirmation occurred in 7/10 cases, as opposed to 1/5 where a diagnosis was unknown pre-test. Thus, diagnostic return is greatest where the diagnosis is known pre-test. For WGS (whole genome sequencing, the next iteration of WES), careful case selection (ideally of known diagnoses pre-test) will yield highest returns.

**Conclusions:**

Our results highlight the cost-effective use of WES-targeted bioinformatic analysis as a diagnostic tool for SD, particularly patients with presumed SD, where detailed phenotyping is essential. Thorough co-ordinated clinical evaluation between clinical, radiological, and molecular teams is essential for improved yield and clinical care. WES (and WGS) yields will increase with time, allowing faster diagnoses, avoiding needless investigations, ensuring individualised patient care and patient reassurance. Further diagnoses will lead to increased information on natural history/mechanistic details, and likely increased therapies and clinical trials.

## Background

Rare diseases by definition affect less than 1 in 2,000 (in Europe) or 1 in 1,500 (in the USA). Approximately 80% of the 7300 rare diseases have a known genetic cause, and whole-exome sequencing (WES) is commonly the final diagnostic step in such cases with diagnostic yields averaging around 31% (24–68%) [1–3].

The broad range in WES diagnostic yields (see Table 1) reflects varying cohorts and clinical indications for testing (i.e., diagnostic rates are higher in selected cohorts with conspicuous phenotypes). Yield is highly influenced by recruitment type (e.g., single proband versus trio), bioinformatic pipelines, study year, consanguinity, case complexity and degree of prior genetic evaluation. Yields tend to increase with study year (due to increasing novel genes, conditions, and technological advances) and in cohorts with a high degree of consanguinity [2]. Conversely yields are lower in cohorts with highly complex cases necessitating challenging interpretation, e.g. cases where two or three independent genetic diagnoses lead to an aggregate phenotype. Yield is also markedly affected by the extent of prior genetic and metabolic evaluation; thus, WES usage earlier in the diagnostic assessment can lead to higher yields.

**Table 1:** Diagnostic yield from NGS strategies in different rare disease cohorts in key publications

Published year	Journal	Strategy	Sequenced	Diagnostic yield (%)	Author
2013	NEJM	WES	250	24.8	Yang et al. [4]
2014	AJHG	WES	264	55.3	Beaulieu et al. [5]
2015	Nat Genet	WGS*	156	21.2	Taylor et al. [6]
2017	JAMA Pediatr	WES	44	52.3	Tan et al. [7]
2017	JAMA Pediatr	WES	278	36.7	Meng et al. [8]
2017	JAMA Pediatr	Critical trio WES	63	50.8	Meng et al. [8]
2018	NPJ Genome Med	WGS*	42	42.9	Farnaes et al. [9]
2019	Sci Rep	WGS*	10	62.5	Liu et al. [3]

*WES* whole-exome sequencing; *WGS* whole-genome sequencing

### Skeletal dysplasia cohorts

The majority of rare diseases (50–75%) affect children [10]. In paediatric unsolved rare disease cohorts, the diagnostic yield from exome sequencing is around 40% [11]. An important subset of rare paediatric disease is skeletal dysplasia (SD). SD conditions are rare disorders of the skeleton, encompassing a genetically heterogeneous group of over 400 distinct disorders. They represent approximately 5% of all congenital anomalies and are a significant contributory cause of children with severe short stature [12].

### Yields in SD

Data on WES yields in SD cohorts are limited. In a large study of over 3000 rare diseases WES cases in a single US centre, Retterer et al., (2015) performed a subgroup analysis of 54 SD cases reporting a diagnostic yield of 39% (n = 54) [13]. Recruitment was predominantly trio based (> 75% were trios, 7% were duos) though the patients were at different points of their diagnostic evaluation. The average patient age was 11 − / + 13 years.

Bae et al. (2015) studied a large cohort of 185 patients with SD across multiple centres in South Korea, who had panel-based targeted exome sequencing (TES) (255 gene panel); 25 had a prior confirmed clinical and molecular diagnosis. Thus, TES was used to reconfirm molecular findings, 64 had an unknown clinical diagnosis, and 96 patients had an assured clinical diagnosis [14]. A molecular diagnosis was ‘confirmed’ or ‘highly pathogenic’ in 20% (n = 13/64) of patients with an unknown clinical diagnosis and 71% (n = 71/96) of patients with an assured clinical diagnosis. In this report, we explore the utility of WES in a cohort solely composed of SD presentations and consider the diagnostic yield in sub-cohorts, i.e., those with an unknown diagnosis and those with an assured diagnosis (either single gene or heterogeneous). Although the number of patients in our study is small (n = 15), to the best of our knowledge, there are no previous reports of a UK/European cohort of patients with SD reported.

## Methods

### Subjects and clinical diagnosis

All patients were study participants at the Guy’s and St Thomas’ Hospital clinical site. Our cohort spans the period; September 2014–2018. All patients within our cohort were paediatric (5 months to 12 years, mean age 5.5 years).

15 patients with presumed SD conditions based on clinical and radiographic findings, were selected from the specialist SD multidisciplinary clinic. The majority of participants were referred to the service from external facilities. Informed consent was obtained for all patients, and thereafter genomic DNA was extracted from the proband and where possible their parents. Many participants were at different stages of their evaluation.

Patients were recruited as trios (proband and both parents) unless otherwise stated.

The 15 patients were divided into three categories based on the certainty of clinical diagnosis and the status of the prospective genotype. Patients with an assured clinical diagnosis of an SD, where one or only a few genotypes are known to be responsible (e.g., achondroplasia) were excluded as they are not appropriate for WES unless single gene testing was not readily available. Patients were then assigned one of three categories.

- Known condition–known gene (Category K–K)
- Unknown condition–unknown gene (Category U–U)
- Known condition/group of conditions–the possibility of multiple genes (Category K–U)

### Exome sequencing and variant prioritisation

WES capture was performed using Agilent SureSelectXT Human All Exon V5 baited with clinically relevant genes followed by sequencing on an Illumina HiSeq 2500 (though three of the later cases were sequenced on an Illumina NextSEQ 550). Raw sequence data were aligned using Novoalign, and variants called with Samtools. Coverage of coding exons (+ / − 5 base pairs) was to a minimum depth of × 20.

### Variant analysis

Qiagen Ingenuity Variant Analysis was used to aid the assessment of variant pathogenicity after applying a virtual panel of 222 SD genes (see Appendix 1) in combination with multidisciplinary clinical interpretation. Qiagen analysis uses (CGI 54 Genomes, SIFT, Exome Variant Server (EVS), Allele Frequency Community, JASPAR, Ingenuity Knowledge Base, Vista Enhancer, BSIFT, TCGA, PolyPhen-2, 1000 Genome Frequency, Clinvar, COSMIC, ExAC, HGMD, PhyloP, DbSNP, TargetScan), and Alamut for splice site analysis (SpliceSiteFinder-like, MaxEntScan, NNSplice, GeneSplicer).

Sequencing data were analysed in three stages. In stage one, clinicians proposed primary genes or small gene-panels for analysis through the WES platform that were likely to harbour the causative variant. This reduced the volume and cost of variants to analyse. If no significant variants were identified, then stage two involved applying and interpreting the 222 SD virtual gene-panel (see Appendix 1). If no appropriate variant was identified in stage two, then stage three involved a human phenotype ontology (HPO) driven whole-exome wide-search. HPO terms were derived by clinical geneticist expert review along with medical record evaluation and blinded radiographic review by three independent SD expert radiologists (CH/AO/AC). The phenotypic terms and differential diagnosis provided were critical for analysis.

### Molecular diagnosis using determined variants

Molecular diagnosis was made by correlating clinical and radiographic findings with candidate sequence variants through WES. The status of the molecular diagnosis was determined as either known or unknown.

## Results

Our cohort of 15 patients comprised: five males and ten females, with ages ranging from 5 months to 12 years with an average age of 5.5 years old. Significant variants were identified in 53.3% (n = 8/15) of patients; 46.7% (n = 7/15) having a definite molecular diagnosis and 6.7% (n = 1/15) having a likely molecular diagnosis. One case (patient 15) after negative WES testing was concluded to have acquired SD aetiology. Thus, 60% (n = 9/15) cases had a confirmed or likely confirmed diagnosis (yield: 60%).

Of unknown clinical diagnosis (n = 5/15), WES led to molecular confirmation (likely or highly likely) in 20% (n = 1/5).

## Discussion

SD diagnoses are challenging for several reasons. Firstly, similar phenotypes are heterogeneous; one gene can cause several conditions. Secondly, the aetiology of multiple SD conditions is unknown or their phenotype not well established. Thirdly, the experience of SD conditions by individual clinicians is limited, and lastly, the characteristic features of many SD conditions only manifest at certain periods. For example, stippled epiphyses (characteristic of chondrodysplasia punctata) are only present in the neonatal or infantile period; thus, diagnoses are difficult without timely investigation. Similarly, many characteristic features disappear after skeletal maturity or only present when young. Multidisciplinary approaches, involving geneticists, radiologists, orthopaedic, growth specialists and therapists (occupational, physiotherapy, psychological), are often necessary for diagnosis and management of SD conditions.

Using a single-gene testing strategy is often unhelpful for the reasons stated, and increasingly WES or WGS options are deployed. In some countries specific panels are used, in place of or prior to WES/WGS, due to lower costs, accessibility and relatively large numbers of genes specific to the phenotype. Infact many WES or WGS approaches begin with panel-based analysis before more agnostic or wider analysis. Little is reported regarding the diagnostic yield of WES in SD conditions, as often such cases are pooled in general rare disease cohorts. Where rates and analysis have been reported, little is discussed around the prior categorisation of cases (e.g., whether the diagnosis was known).

Despite these challenges, yields for SD conditions are relatively high compared to other rare disease categories. In fact, for fetal anomalies, the highest yields (~ 80%) were obtained in the SD category in the UK fetal exome PAGE (prenatal assessment of genomes and exomes) study [15]. However, the yield is less spectacular postnatally. Retterer et al. report a yield of 39% in SD patients (n = 54) using WES in a combined paediatric and adult cohort [13]. Bae et al., reported a likely or confirmed molecular diagnosis in 52.5% (n = 84/160) through TES, though the patient ages are not reported [14].

To diagnose SD conditions, radiographic imaging in early childhood is key; thus, our cohort of only paediatric patients consisted of suitably appropriate clinical-radiographic data for further genetic investigation. None of the patients had molecular confirmation of their condition before WES testing. Upon WES testing, a three-stage approach to variant analysis (as outlined in the methods) was undertaken. This allowed molecular scientists to focus their search, identifying pathogenic variants efficiently and saved time spent analysing less likely variants.

Significant variants leading to a likely or confirmed diagnosis were identified in 53.3% (n = 8/15) of patients; 46.7% (n = 7/15) having a definite molecular diagnosis and 6.7% (n = 1/15) having a likely molecular diagnosis. In comparison, Bae et al. reported a likely or confirmed molecular diagnosis in 52.5% (n = 84/160) of similar patients (excluding those patients who had molecular confirmation prior to WES testing) [14]. This means that our yield was comparable to Bae et al., and was marginally higher. If we include patient 15 as a confirmed diagnosis, who after negative WES testing was concluded to have an acquired SD aetiology, then our yield reached 60% (n = 9/15).

The possible reasons for a slightly higher yield as compared to Bae et al. includes increased gene discovery linked to SD conditions since Bae et al. was published (2016), improved bioinformatic pipelines, technological advances in next-generation sequencing machinery (e.g. improved coverage and read depth) and triple radiological review within our analysis (all three reviewers being expert contributors to the International Skeletal Dysplasia Society) [14]. We note that radiological input was key with patients 2 and 4. For patient 2, the radiology review led to a change in the pre-test working diagnosis from spondyloepiphyseal dysplasia congenita (SEDC) to campomelic dysplasia (CD), enabling a better genotype–phenotype match, thus securing the diagnosis. Likewise, the radiological diagnosis with patient 4 identified the correct spectrum of disorders so that when a *COL11A2* (collagen type XI alpha-2 chain) variant was found, the appropriate final diagnosis was more easily identified.

Of patients with a known diagnosis pre-WES (n = 10), WES led to a confirmed molecular diagnosis in 7/10 cases (rising to 8/10 if we include patient 8 as another confirmed diagnosis, though of an acquired cause). This highlights the significant return when there is a known diagnosis pre-test.

Of patients with unknown clinical diagnoses (n = 5/15), WES led to molecular confirmation (likely or highly likely) in 20% (n = 1/5) of cases which is the same detection rate as Bae et al. [14] This was expected considering low pre-test diagnostic SD hypotheses.

WES yields for non-SD conditions (25–51%, see Table 1) are generally lower than for SD disorders (this study; up to 60% and Bae et al. 52.5%) [14]. This is likely due to the rich combination of radiology and clinical features, and synergism between multiple experts, enhancing phenotypic-driven bioinformatics analysis.

We further discuss selected patients to extrapolate key learning points and have grouped the patients into themes.

### Theme 1: known clinical diagnosis with clear single-gene cause, but testing unavailable

Patient 1 had a clinical diagnosis of spondyloepiphyseal dysplasia tarda (SEDT; OMIM #313,400). SEDT is caused by heterozygous *TRAPPC2 (*tracking protein particle complex subunit 2) variants. Several males across multiple generations had a similar diagnosis but no known genetic testing. At the time of diagnosis in patient 1 (2014), *TRAPPC2* testing was not readily available in the UK (except on a research basis) [16]. Thus, WES was performed, with primary analysis directed towards *TRAPPC2*. A pathogenic TRAPPC2 variant was identified (see Table 2). With many laboratories moving to WES and WGS, the availability of many single-gene tests is decreasing; thus, WES approaches with targeted analysis are becoming ubiquitous. It is therefore crucial that clinicians make a pre-test diagnosis to enable targeted analysis and prevent the generation of unwanted and unrelated variants.

**Table 2:** Summary of patients with categorisation, features, and pre- and post-testing diagnosis

Patient	Sex	Age	Category	Clinical presentation	Variants	Inheritance Studies	Revised diagnosis
1	M	11y	K–K	Spondyloepiphyseal dysplasia tarda X- Linked	Hemizygous deletion of *TRAPPC2* exon 6	Maternally inherited	Spondyloepiphyseal dysplasia tarda (X-Linked)†
2	F	10y	K–K	Spondyloepiphyseal dysplasia congenita	Heterozygous *SOX9* c.508C > T, p.(Pro170Ser) variant detected	De-novo variant not present in parents	Surviving campomelic dysplasia (CD)†
3	F	5 m	K–K	Syndromal proportional extreme short stature, Chiari malformation type I, growth hormone deficiency, hydrocephalus. Suspected Laron syndrome	No pathogenic variants were detected	Not applicable	SD of unknown aetiology‡
4	F	0-1y	K–U	Mild fibrochondrogenesis type 2. Post radiological review, changed to severe otospondylomegaepiphyseal dysplasia	Heterozygous *COL11A2* c.2542C > T p.(Gln848Ter) variant detected (mat) Heterozygous *COL11A2* c.3151-2delA variant detected (pat))	AR—one variant inherited from each parent—trans	Severe otospondylomegaepiphyseal dysplasia†
5	F	6y	K–U	Multiple epiphyseal dysplasia with non-specific myopathy, or metaphyseal dysplasia	Heterozygous *MATN3* c.400G > A p.(Glu134Lys)	Variant not present in mother. Father not tested	Multiple epiphyseal dysplasia†
6	F	8y	K–U	Intrauterine growth restriction (IUGR), failure to thrive, developmental delay, microcephaly, dysmorphisms, short stature. Type of microcephalic osteodysplastic primordial dwarfism	Heterozygous *PCNT* c.5812C > T p.(Gln1928Ter) variant detected Heterozygous *PCNT* c.9273 + 1G > C variant detected	AR—one variant inherited from each parent—trans	Microcephalic osteodysplastic primordial dwarfism type II†
7	M	3y	K–U	Metaphyseal chondromatosis with D2-hydroxyglutaric aciduria	Heterozygous *IDH1* c.395G > A p.(Arg132His) variant detected	De novo—variant not present in parents	Metaphyseal chondromatosis with D2-hydroxyglutaric aciduria†
8	F	2y	K–U	Chondrodysplasia punctata. Query genetic or teratogen	No pathogenic variants were detected	Not applicable	Possible acquired aetiology§
9	F	11y	K–U	AR osteopetrosis (infantile onset)	Heterozygous *CLCN7* c.1468delC p.(Leu490fs) variant detected (pat) Heterozygous *CLCN7* c.1853C > A p.(Ala618Asp) variant detected (mat), VUS	AR—one variant inherited from each parent—trans	AR osteopetrosis type 4 (infantile onset) †
10	M	12y	K–U	Acromesomelic dysplasia, Maroteaux type, or spondylarperipheral	No pathogenic variants were detected	Not applicable	SD of unknown aetiology‡
11	F	3y	K–U	Odontochondrodysplasia	No pathogenic variants were detected	Not applicable	Odontochondrodysplasia¶
12	M	1y	U–U	Disproportionate short stature, conductive hearing loss, percutaneous endoscopic gastrostomy fed, dysmorphisms	No pathogenic variants were detected	Not applicable	SD of unknown aetiology‡
13	F	2y	U–U	SD; congenital heart disease, hemimegaloencephaly, bilateral iris colobomata, hearing loss	Heterozygous *EXT2* c.237G > A p.(Trp79Ter) variant detected (mat) Heterozygous *EXT2* c.1404 + 2 T > C variant detected (pat)	AR—one variant inherited from each parent—trans	Autosomal recessive exostosin glycosyltransferase 2 syndrome^
14	M	7y	U–U	Query EDS (classic type/collagenopathy), or Mandibular acrodysplasia type A	No pathogenic variants were detected	Not applicable	SD of unknown aetiology‡
15	F	6y	U–U	Unknown SD, rule out bone marrow transplant-related short stature	No pathogenic variants were detected	Not applicable	Bone marrow transplant-related short stature§

^†^Confirmed molecular diagnosis

^‡^Unknown diagnosis

^§^Possible acquired diagnosis

^¶^Confirmed clinical diagnosis but nil molecular findings

^Possible molecular diagnosis

*K–K* known condition–known condition; *K–U* known condition–unknown condition; *U–U* unknown condition–unknown condition; *SD* skeletal dysplasia; *AR* autosomal recessive; *TRAPPC2* tracking protein particle complex, subunit 2; *SOX6* sex determining region Y-box 6); *COL11A2* collagen type XI alpha-2 chain; *MATN3* matrilin-3; *PCNT* pericentrin; *IDH1* isocitrate dehydrogenase 1; *CLCN7* chloride voltage-gated channel 7; *VUS* variant of unknown significance; *EXT2* exostosin glycosyltransferase 2; *EDS* Ehlers–Danlos syndrome

### Theme 2: known clinical diagnosis (heterogeneous condition), multiple potential genetic causes

**Fig. 1:**
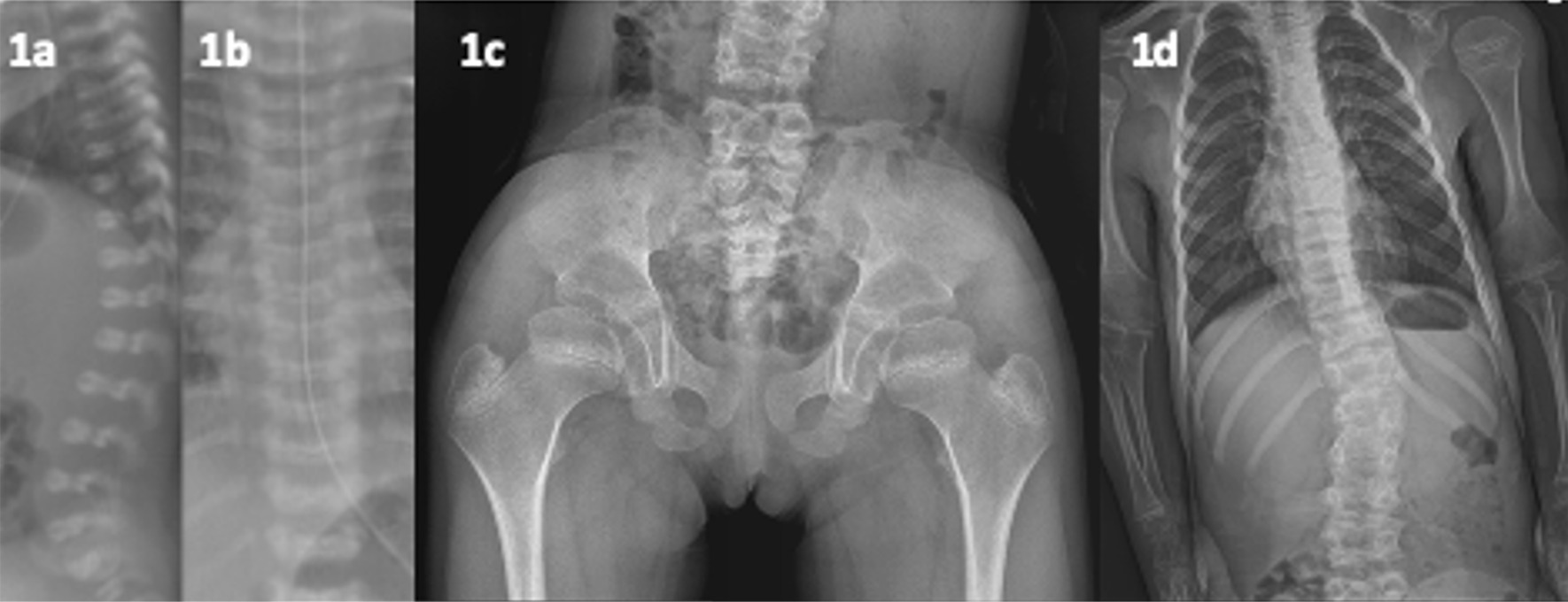
(patient 4, OSMED). **a** and **b** Age day 3; sagittal spinal clefts, coronal spinal clefts. **c** and **d** age 6 years; mega-epiphyses of the femoral head, wide metaphyses, C-shaped left convexity of the spine, marked anterior wedging of T11, T12 and L1

Patient 4 had macrocephaly, a flat facial profile, nasal bridge depression, small nose, micrognathia, a small bell-shaped thorax and short long-bones with widened metaphyses. The original diagnosis was ‘mild’ fibrochondrogenesis type 2 (FBCG2; OMIM #614524), which is caused by *COL11A2* variants and can be lethal. The differential included fibrochondrogenesis type 1 (OMIM #228520) caused by *COL11A1* as well as Stickler syndrome (OMIM #108300) hence multiple genes could be potentially causative; thus, WES was an appropriate strategy. The radiological review noted vertebral coronal and sagittal clefts and with increasing age (see Fig. 1); enlarged epiphyses and clinically hearing loss became manifest. This led to an alteration of the diagnosis to otospondylomegaepiphyseal dysplasia (OSMED; OMIM #215150), an allelic disorder to FBCG2. WES confirmed compound heterozygous *COL11A2* variants which cause a spectrum of disorders from mild deafness to OSMED to potentially lethal FBCG2. This case illustrated radiological (e.g. mega-epiphyses) and clinical clues (hearing loss) that may only become apparent with time. It also highlights the need for clinicians to be careful of using ‘old’ diagnostics labels as milder forms of previously ‘lethal’ conditions or what we considered ‘extremely’ severe phenotypes are emerging. In such instances, umbrella terms like *COL11A2*-spectrum disorder as a pre-molecular diagnosis may be more apt rather than FBCG2.

Patient 5, a six-year-old male of normal stature who presented with leg pain, genu valgum, pes planus, muscle weakness and decreased hip mobility. His clinical picture and radiology suggested possible multiple epiphyseal dysplasia (MED; OMIM #132400) with non-specific myopathy. Creatinine kinase was normal, and electromyography suggested myopathic disorder, muscle biopsy showed mild type 2 fibre atrophy and array Comparative Genomic Hybridisation (aCGH) was normal. MED is characterised by multiple long and or short bone epiphyseal abnormalities and has autosomal dominant (ADMED; OMIM #132400) and autosomal recessive (ARMED; OMIM #226900) forms [17, 18]. ADMED can be due to; *COMP* (cartilage oligomeric matrix protein)*, COL9A1-COL9A3* (collagen type IX alpha 1–3 chain) *or MATN3* (matrilin 3) and presents in childhood with joint pain, exercise-induced fatigue, restricted mobility, short stature, and early-onset osteoarthritis [18]. ARMED is caused by *SLC26A2 (*solute carrier family 26, member A2) formerly known as *DTDST* (diastrophic dysplasia sulfate transporter) and presents with joint pain and often mild short stature. *SLC6A2* is responsible for three allelic skeletal dysplasias; (in increasing severity) diastrophic dysplasia, atelosteogenesis 2 and achondrogenesis type 1B [17]. Further key ARMED differentials include; mild mucolipidosis III (OMIM #252605) and mucopolysaccharidosis VI (OMIM #253000), both recessive storage disorders discriminated from ARMED by coarse dysmorphic facies, intellectual disability, visceral involvement, marked spondylar disease and shorter stature [1].

**Fig. 2:**
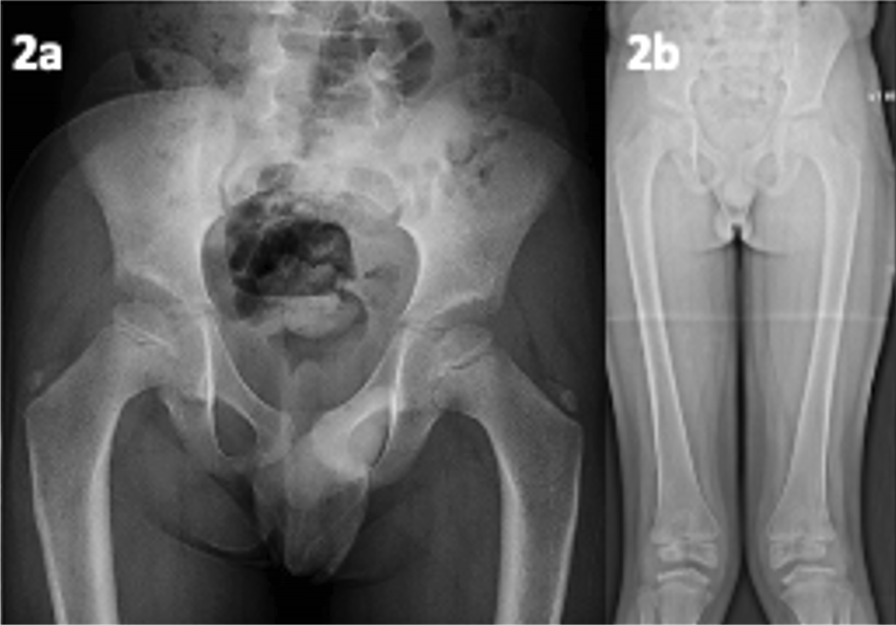
(patient 5, MED). **a** Age 7 years; bilateral femoral head epiphyseal dysplasia, medial aspects fragmented. **b** Age 11 years; genu valgum and underdeveloped distal medial femoral epiphyses

Radiology showed delayed epiphyseal (long-bone) ossification and small and irregular epiphyses (especially in the knees and hips) (see Fig. 2). Due to MED heterogeneity, WES was an appropriate strategy for molecular diagnosis and identified a pathogenic *MATN3* variant confirming MED Type 5 (OMIM #607078).

**Fig. 3:**
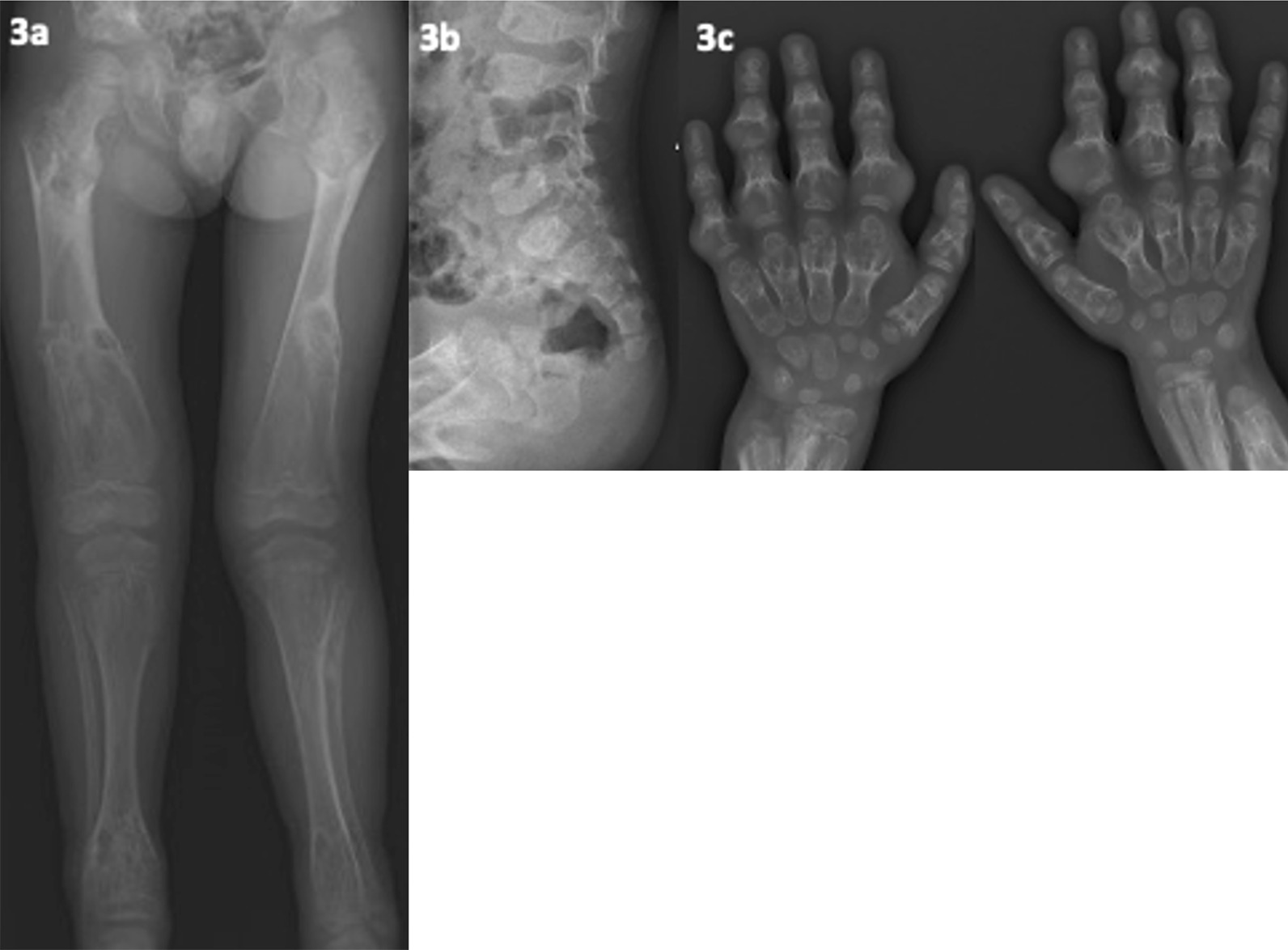
(patient 7, metaphyseal chondrodysplasia—D2-HGA type). **a** Age 7 years; left leg is longer than right, marked left-sided genu valgum. Multiple expansile lucent metaphyseal lesions involving bilateral femora and tibia. **b** Age 2.5 years; expansion and irregularities of vertebral bodies. **c** Age 7 years; extensive enchondromatosis of both hands and wrists with associated soft tissue swelling

Patient 7 presented aged 3 years with an undefined SD, multiple falls, complex congenital heart disease (CCHD), relative macrocephaly and mild developmental delay. Skeletal survey at 10 months highlighted irregular metaphyses. He had leg-length discrepancy, right leg bowing, mild joint hypermobility and hypotonia. Further radiology (Fig. 3a, b) revealed worsening metaphyseal dysplasia (expansion, cup-shaped irregularity) with radiolucent non-ossified cartilage affecting major joints. At 6.5 years, hand radiology showed severe widespread bilateral multiple enchondromas (see Fig. 3c), that were also present in the lower limbs (see Fig. 3a). The diagnosis was felt to be an enchondromatosis-like condition and upon radiological review; metaphyseal chondromatosis with D-2-hydroxyglutaric aciduria (MC-D2HGA; OMIM #614875) was suspected. Though MC-D2HGA was highly suspected, other genetic disorders with very similar phenotypes remained thus WES was an appropriate strategy.

**Fig. 4:**
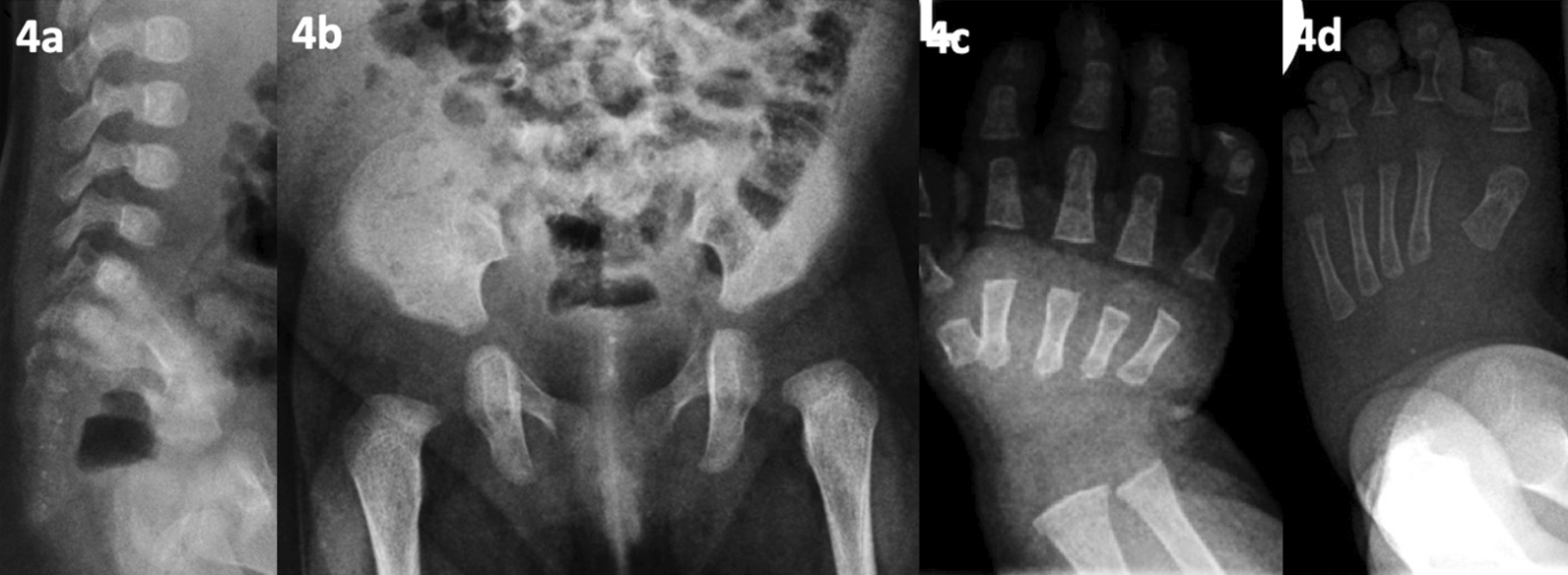
(patient 8, acquired SD). Aged 2 months; **a** sacral stippling, small, round/ovoid vertebral bodies with coronal clefts in the thoracic spine. **b** Sacral stippling. **c** short first metacarpals. **d** stippled epiphyses distal to the tibia and delayed ossification of the tarsal bones

Enchondromatosis (EC) is a rare heterogeneous condition with multiple enchondromas (benign hyaline cartilage forming tumours in the metaphysis) [19]. The commonest two subtypes are Ollier disease (OMIM #166000) and then Maffucci syndrome (OMIM #614569) [19]. MC-D2HGA is another rarer subtype with only 11 reported cases (four of which are due to somatic mosaicism of *IDH1* (isocitrate dehydrogenase 1)*,* the remainder lacking molecular confirmation) (Fig. 4) [20].

WES testing identified a de novo heterozygous *IDH1* c.395G > A p.(Arg132His) germline variant (approximately indicative allele frequency of 50/50, wild type: a variant on Sanger). Urinary organic acid testing showed marked isolated increase in 2-hydroxyglutarate, confirming MC-D2HGA (this result came after WES was initiated but prior to the WEST results). MC-D2HGA is associated with macrocephaly, developmental delay, hypotonia, significant metaphyseal dysplasia, enchondromatosis, dysmorphia and CCHD. This is the first reported MC-D2HGA case caused by germline *IDH1* changes (with read depth over the IDH1 variant position of >  × 250 depth) though the author is aware of one unreported MC-D2HGA case caused by germline changes (Zankl A. [Presentation] 6th Nordic Workshop on Skeletal Dysplasia, Karolinska Institute, Sweden. 5th March 2020.) Had a WES approach not been used, it would have been difficult to reach this diagnosis.

**Fig. 5:**
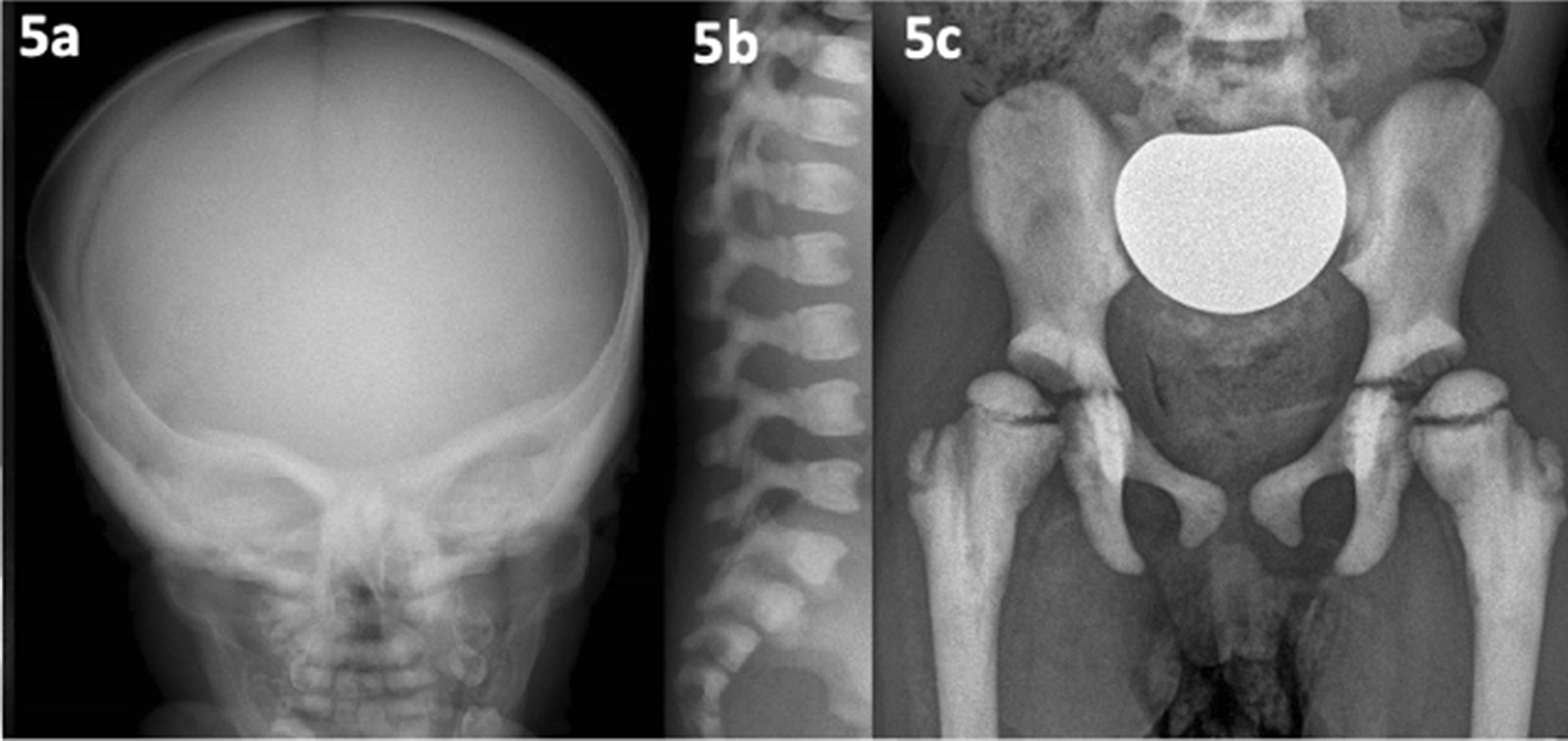
(patient 9, infantile osteopetrosis). **a** and **b** Aged 1 year; thickened cortex skull, bone-in-bone appearance of vertebrae, anterior concavity. **c** Age 8 years; sclerotic pelvic bones/femora

Patient 9 presented with osteopetrosis, a heterogeneous condition, classically divided into; fatal infantile malignant form (OMIM #259700/611490/259720), Albers-Schonberg disease (OMIM #166600/259700) and a milder adult form. She presented with facial nerve palsy, and subsequent radiography noted Rickettsial-like bony ends (see Fig. 5). Foraminal impingement led to hearing loss and visual problems and a working diagnosis of infantile-onset osteopetrosis was established, suitable for WES. The proband had compound heterozygous variants in *CLCN7* (chloride voltage-gated channel 7); a pathogenic variant inherited from her unaffected father and a VUS (variant of unknown significance) from her unaffected mother, though her maternal grandmother was affected (late-onset osteopetrosis) and had the same VUS. It later transpired that the mother actually had mild radiographic and clinical features demonstrating segregation and striking intrafamilial variable expression of osteopetrosis type 4 (OMIM #611490). If PP4 (protein phosphate-4) or PP1 (protein phosphate-1) panels were applied, the VUS would be upgraded to likely pathogenic. The paternal variant alone did not manifestly cause disease in the father but was felt to negatively modify the severity of the condition in the presence of the maternal variant.

### Theme 3: known diagnosis, no known gene

**Fig. 6:**
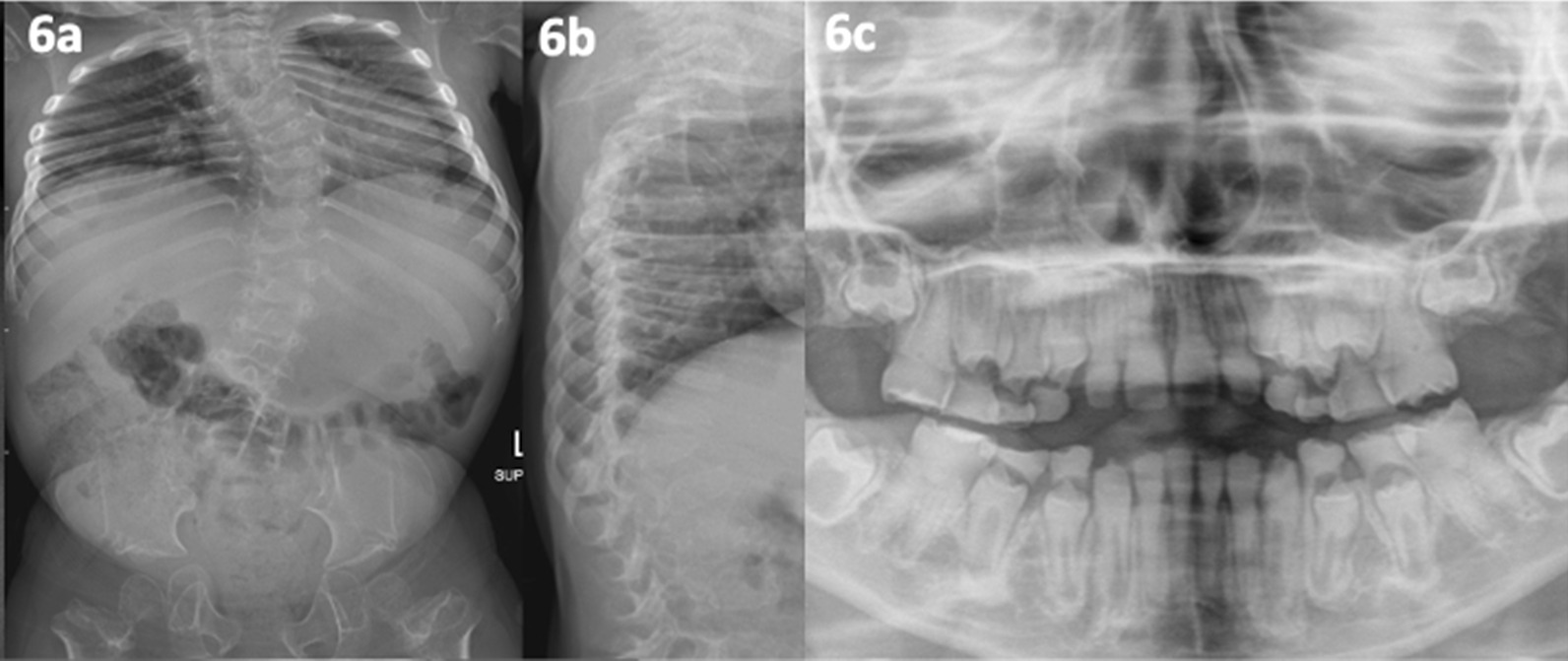
(patient 11, odontochondrodysplasia). **a**, **b** Aged 5.5 years; severe s-shaped scoliosis, marked platyspondyly, segmentation abnormality at T4/5, spondylolisthesis, slender ribs posteriorely with mild anterior rib flaring, proximal femoral metaphyseal dysplasia. **c** 3 years dentine dysplasia

In 2015, Patient 11, aged 3,presented with severe short stature, delayed motor milestones, a high-pitched voice, dysmorphism, ligamentous laxity, instability of the cervical spine, severe scoliosis (see Fig. 6a, b), discoloured/weak dentition (see Fig. 6c), and no fractures. The working diagnosis after radiological review was a rare form of ‘spondylometaphyseal dysplasia with dentinogenesis imperfecta, odontochondrodysplasia (OMIM #184260). At the time, this condition had no known gene cause. Thus, an agnostic approach via WES was appropriate for new gene discovery. No pathogenic variants were found. Some years later, a causative gene was discovered for the condition, *TRIP11*(thyroid receptor-interacting protein 11) though despite re-analysis, a molecular cause was not confirmed in patient 11. Other genetic causes of odontochondrodysplasia are still sought.

### Theme 4: unknown SD diagnosis

Patient 13 presented with SD (short stature, macrocephaly, and scoliosis) with non-skeletal syndromal features (congenital heart disease and hearing loss). *CHD7* (chromodomain helicase DNA binding protein 7) gene testing for CHARGE syndrome (OMIM #214800) was negative. WES identified two likely pathogenic variants in *EXT2* (exostosin glycosyltransferase 2) found in *trans* with clinical findings overlapping for AREXT2 syndrome (autosomal recessive exostosis-2 gene syndrome). AREXT2 is an ultra-rare recessive disorder with an unclear and expanding phenotype as only four families have been reported [21]. First described in 2015, it was termed Seizures, Scoliosis and Macrocephaly syndrome (SSM; OMIM #616682). Later authors suggested the ‘AREXT2’ label to recognise the lack of uniformity of scoliosis or seizures. It is unclear whether patient 13 has AREXT2 thus, functional work is required. In this unknown syndromal case, WES has ruled out many potential diagnoses and the WES-based agnostic approach allowed for further investigation of an emerging ultra-rare condition.

### Theme 5: WES to exclude a genetic diagnosis and support an acquired cause

Chondrodysplasia punctata (CDP) is a rare SD characterised by punctiform calcification of cartilage and is acquired or genetic in origin [22]. Genetic forms (OMIM #302950/118650) are heterogeneous. Acquired forms can be due to maternal malabsorption of vitamin K, maternal warfarin or some anticonvulsant [23]. Patient 8, a 2-year-old female, presented with faltering growth and antenatal exposure to lamotrigine and topiramate. Antenatal scans demonstrated short long-bones. Post-natal radiology (see Fig. 4a–d) showed sacral stippling with delayed tarsal ossification, consistent with CDP [24]. Since several genes cause CDP, WES is an appropriate strategy to reasonably exclude underlying genetic causes. Thus, negative WES increases antenatal anticonvulsant exposure as the likely cause. Although there is no reported association of lamotrigine and topiramate, causing stippling, other Hydantoin anticonvulsants (e.g. phenytoin) have been reported to do so [23]. This case highlights the need for early radiography, as stippling is often only seen in the first year of life and rarely after age three [25].

**Fig. 7:**
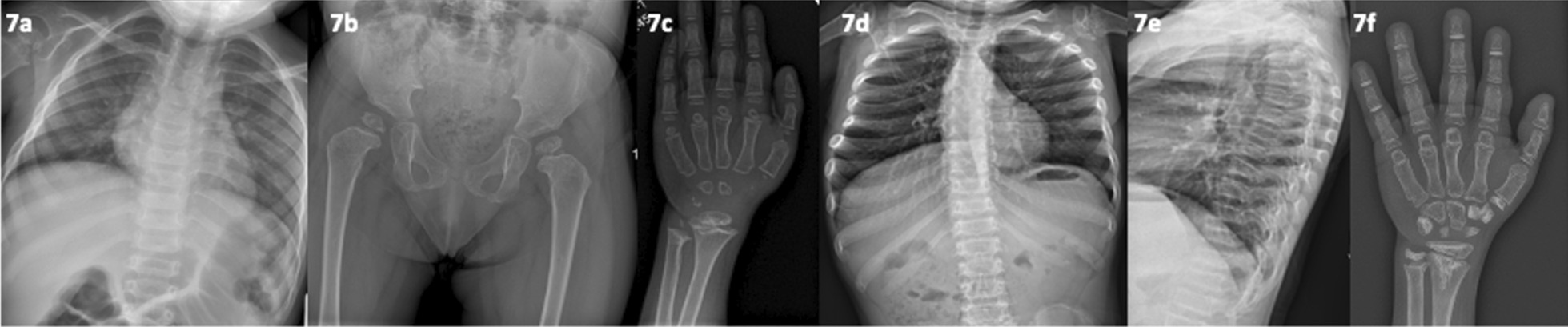
(patient 15, post HSCT related SD). **a–c** Aged 5 years; broad ribs, platyspondyly, proximal femoral epiphyseal dysplasia/metaphyseal irregularity. **d–f** Age 8 years; progressive changes; broadening of ribs, worsening vertebral end-plate irregularities, diffuse platyspondyly, increased density of carpal bones (excluding the hamate and capitate),increase phalangeal epiphyseal density, dense distal ulnar with v-shaped chondroid type calcification extending distally

Patient 15 was born with normal birth and growth parameters until age 11 months when an unexplained prolonged fever led to a diagnosis of haemophagocytic lymphohistiocytosis (OMIM #603553). Biallelic *PRF1* (Perforin-1) pathogenic variants (compound heterozygote) were confirmed. At 18 months, matched unrelated cord blood HSCT (homologous stem cell transplantation) was performed. Subsequent progressive growth failure developed, associated with radiographic spondyloepimetaphyseal chondrodysplasia (progressive changes as shown in Fig. 7a–f, note radiographs prior to HSCT were not take as skeletal dysplasia was not suspected then), functional asplenia and sensorineural hearing loss. Development of skeletal changes throughout childhood including fixed flexion hip deformity, marked pes planus, marked genu valgum and pectus carinatum. Extensive endocrine investigations returned no cause. WES (and subsequent WGS) was negative for a skeletal cause. An international group of SD experts noted the similarity of this case and several others, concluding a potentially new disorder manifesting with growth failure and a chondrodysplasia phenocopy post early HSCT for non-oncological disorders [26]. This case highlights the utility of WES to reduce the likelihood of a genetic cause significantly and increase the confidence of an acquired cause [26].

### Further discussion

As many previous studies have discussed, a broad approach to genetic testing through WES or WGS allows for the identification of conditions that may not have been suspected clinically and thus the expansion of previously known phenotypes.

In our cohort of 15 patients, the use of WES has led to three novel findings. Firstly, Patient 7 is the first reported case of MC-D2HGA due to a germline variant. Secondly, patient 13, is potentially the fifth reported family with AREXT2 syndrome providing further expansion of the phenotype. Lastly, patient 15, is one of seven patients who have collectively provided evidence for a new disease, ‘chondrodysplasia phenocopy post early HSCT for non-oncological disorders’ and has been submitted for publication [26].

Although WES remains the current ‘go-to’ diagnostic test in many rare disease scenarios, we increasingly see a shift to WGS, especially for the acutely ill child. Even then, a large body of undiagnosed patients remain. When WES returns negative or inconclusive results, for many, the diagnostic odyssey is abandoned or halted. Yet it is clear that re-analysis of WES data can often result in diagnosis in 10–15% of these cases [2].

Additional testing such as long read sequencing (LRS), copy number variants (CNV) in non-coding regions, non-coding variants (NCV), repeat expansion (RE), methylation testing (MT) and other structural changes will need to be explored to increase diagnostic yield. Burdick et al. explored the proportion of diagnoses from additional testing in 54 patients with clinical diagnoses, enrolled in their Undiagnosed Disease Network [2]. Of the 54 participants, the molecular diagnosis was obtained in 36 (67%) through WES, and in 15 (28%) through additional testing. Of these, 7/15 (47%) had an NCV, 6/15 (40%) a CNV, and 2/15 (13%) had a RE or a DNA methylation disorder. A yield figure could not be given since there were many other patients within the programme, who were at different stages of assessment. Nevertheless, the report highlights the benefit of testing beyond WES and the approaches that can be considered.

This combined with the advance of radiomics (the systematic use of artificial intelligence to provide diagnostic processing and analysis of ever sophisticated imaging data) will usher in an increased yield. The careful combination of the physician (endocrinologist, orthopaedic surgeon), radiologist and geneticist working together will be crucial.

The difficulty with large scale sequencings, such as WES or WGS is the generation of large numbers of variants of unknown significance (VUS), especially as genetic testing, is mainstreamed. Likewise, bioinformatic pipelines can present de-novo variants with strong computational evidence suggestive of particular conditions, that are not easy to dismiss. The present challenge is, therefore, that of ‘variant interpretation’. Further familial segregation is not always possible.

Traditionally, one way to assess a VUS is to perform functional studies (e.g. fresh blood samples to undertake RNA studies to see if the DNA change has a functional impact on RNA production and therefore on the given protein). This method is challenging as it requires fresh blood samples (as RNA degrades quickly), a difficulty, especially in the paediatric population and blood samples are not always the appropriate sample to test a particular variant as the gene of interest may not particularly be expressed.

Additionally, RNA studies are not always possible in the NHS diagnostic lab, and clinicians often had to partner with university academics or other institutions for functional work (often by providing fresh blood samples, or skin samples or saliva). The problems with this are manifold; time-intensive and dependent on knowing suitable partners. Furthermore, obtaining the required sample can be difficult; we have already discussed the difficulty with blood samples. Skin samples require a skin biopsy which is a relatively invasive procedure. Saliva samples are often difficult to work with, of poor quality/limited in what can be assessed. Lastly, with more and more functional study requests to academics, the boundaries of responsibility for what are essentially research analyses are blurred, thus increasing reluctance from academia to perform such work (culpability issue). Suitable new ‘quick and easy’ tests need developing for variant analysis, and hair pluck analysis may be one such avenue in the SD domain, due to beneficial expression profiles of SD genes.

## Conclusion

Although the genetic causes of > 450 forms of skeletal disorders have been rapidly uncovered, distinct SD conditions of unknown genetic aetiology remain [27]. The best strategy for identifying these may be the unbiased approach of WES or WGS, especially in children and patients with severe or multi-systemic diseases. It can be particularly beneficial to detect de novo pathogenic variants using a trio design. In sporadic cases, analysis of trios may reveal de-novo pathogenic variants. In familial cases, a combination of WES and either WGS or SNP typing could yield linkage information for prioritisation of rare variants [28]. If no variants are identified through WES, then further testing is needed, though the cost-effectiveness of such studies needs determination [2].

Our results highlight the cost-effective use of WES-targeted bioinformatic analysis as a diagnostic tool for SD, particularly for patients with presumed SD, where detailed phenotyping is essential. The thorough clinical evaluative approach and planning between clinical, radiological, and molecular teams is essential for improved service provision.

As we move towards WGS trio sequencing, experience and this study shows that careful categorisation of patients of those with known pre-test diagnosis will yield a higher return of molecular confirmation (8/10 in this study) as opposed to unknown diagnosis pre-test (1/5 in this study) thus highlighting the importance of carefully selecting the most suitable cases for highest WGS yield. With the evolution of this pilot study, the SD panel was extended to 498 genes and anecdotally several more pre-test unknown SD cases were molecularly resolved, thus it is felt WES/WGS will play a more significant role in cases with no prior test diagnosis.

The clinical yield of WES will increase over time, allowing providers to arrive at a diagnosis efficiently. This information will help avoid needless diagnostic procedures/costly additional tests and individualised patient care, allowing for informative clinical decisions and reassurance to patients and families [13]. More diagnoses will, in turn, lead to more information on natural history, improved mechanistic details, and hopefully increased therapies and clinical trials.

## Data Availability

The datasets generated during and/or analysed during the current study are not publicly available due to maintaining patient confidentiality but are available from the corresponding author on reasonable request.

## Appendix 1: The skeletal dysplasia gene panel v1 (222 genes, ‘Pan 272’) in accordance with UK Genetic Testing Network

ACAN, ACP5, ACVR1, ADAMTSL2, AGPS, ALPL, ALX1, ALX3, ALX4, ANKH, ANO5, ANTXR2, ARHGAP31, ARSE, ATP6V0A2, B3GALT6, B4GALT7, BMP1, BMP2, BMPR1B, CA2, CANT1, CASR, CC2D2A, CCDC8, CDC6, CDH3, CDKN1C, CDT1, CEP290, CHST14, CHST3, CHSY1, CLCN5, CLCN7, COL10A1, COL11A1, COL11A2, COL1A1, COL1A2, COL2A1, COL9A1, COL9A2, COL9A3, COMP, CREB3L1, CRTAP, CTSK, CUL7, DDR2, DHCR24, DLL3, DLX3, DMP1, DYM, DYNC2H1, EBP, EFNB1, EFTUD2, EIF2AK3, ENPP1, ESCO2, EVC, EVC2, EXT1, EXT2, FAM20C, FBLN1, FBN1, FBXW4, FERMT3, FGF10, FGF23, FGF9, FGFR1, FGFR2, FGFR3, FKBP10, FLNA, FLNB, FMN1, FOXC1, GALNT3, GDF5, GLI3, GNAS, GORAB, GPC6, GPX4, GREM1, HDAC4, HOXA11, HOXD13, HPGD, HSPG2, ICK, IFITM5, IFT122, IFT140, IFT172, IFT43, IFT80, IFT88, IHH, IKBKG, IL1RN, INPPL1, KAT6B, KIF22, KIF7, LBR, LEMD3, LEPRE1, LFNG, LIFR, LMBR1, LMNA, LMX1B, LRP4, LRP5, MAFB, MATN3, MESP2, MGP, MKS1, MMP13, MMP2, MMP9, MSX2, MYCN, NEK1, NIPBL, NKX3-2, NLRP3, NOG, NOTCH2, NPR2, NSDHL, OBSL1, OFD1, ORC1, ORC4, ORC6, OSTM1, PAPSS2, PCNT, PEX7, PHEX, PIGV, PITX1, PLEKHM1, PLOD2, POLR1C, POR, PPIB, PRKAR1A, PTDSS1, PTH1R, PTHLH, PTPN11, PYCR1, RAB23, RASGRP2, RECQL4, ROR2, RPGRIP1L, RUNX2, SALL1, SALL4, SBDS, SERPINF1, SERPINH1, SH3BP2, SH3PXD2B, SHH, SHOX, SLC25A12, SLC26A2, SLC34A3, SLC35D1, SLC39A13, SMARCAL1, SOST, SOX9, SP7, SULF1, TBCE, TBX15, TBX3, TBX4, TBX5, TBXAS1, TCIRG1, TCOF1, TCTN3, TGFB1, THPO, TMEM216, TMEM38B, TMEM67, TNFRSF11A, TNFRSF11B, TNFSF11, TP63, TRAPPC2, TREM2, TRIP11, TRPS1, TRPV4, TTC21B, TWIST1, TWIST2, TYROBP, WDR19, WDR34, WDR35, WDR60, WISP3, WNT3, WNT5A, WNT7A, ZMPSTE24.

## Abbreviations

aCGH: Array comparative genomic hybridisation
ACH: Achondroplasia
AMD: Acromesomelic dysplasia
AR: Autosomal recessive
AREXT2: Autosomal recessive exostosin glycosyltransferase 2
CCHD: Complex congenital heart disease
CD: Campomelic dysplasia
CDP: Chondrodysplasia punctata
CHD7: Chromodomain helicase DNA binding protein 7
CLCN7: Chloride voltage-gated channel 7
COL11A2: Collagen type XI alpha-2 chain
COL2A1: Collagen type II alpha-1 chain
COL9A1-COL9A3: Collagen type IX alpha 1–3 chain
COMP: Cartilage oligomeric matrix protein
DD: Developmental delay
EC: Enchondromatosis
EDS: Ehlers–Danlos syndrome
EVS: Exome variant server
EXT2: Exostosin glycosyltransferase 2
FBCG2: Fibrochondrogenesis type 2
GH: Growth hormone
HPO: Human phenotype ontology
HSCT: Homologous stem cell transplantation
IDH1: Isocitrate dehydrogenase 1
IUGR: Intrauterine growth restriction
MATN3: Matrilin-3
MC-D2HGA: Metaphyseal chondromatosis with D-2-hydroxyglutaric aciduria
MED: Multiple epiphyseal dysplasia
MPOD: Microcephalic osteodysplastic primordial dwarfism
OSMED: Otospondylomegaepiphyseal dysplasia
PAGE: Prenatal assessment of genomes and exomes
PEG: Percutaneous endoscopic gastrostomy
PCNT: Pericentrin
PRF1: Perforin-1
PP1: Protein phosphate-1
PP4: Protein phosphate-4
SD: Skeletal dysplasia
SEDC: Spondyloepiphyseal dysplasia congenita
SEDT: Spondyloepiphyseal dysplasia tarda
SOX-9: SRY (sex determining region Y)-Box 9
SSM: Seizures, scoliosis and macrocephaly syndrome
TES: Targeted exome sequencing
TRAPPC2: Tracking protein particle complex, subunit 2
TRIP11: Thyroid receptor-interacting protein 11
VUS: Variant of unknown significance
WES: Whole-exome sequencing
WGS: Whole-genome sequencing
100kGP: 100,000 Genomes project
